# The Camille Bernard Flap for Lower Lip Reconstruction

**Published:** 2015-06-26

**Authors:** Adrian Frunza, Slavescu Dragos, Amit Beedasy, Oana Grobnicu, Ioan Lascar

**Affiliations:** Bucharest Emergency Clinical Hospital, Bucharest University School of Medicine, Romania

**Keywords:** lip reconstruction, lip defects, Camille Bernard flap, oral competence, lip tumor

## DESCRIPTION

A 69-year-old man presented to our clinic for a lower lip tumor that developed over the last 4 years. It extended to about 4 cm in length and 4 cm in height involving the full thickness of lower lip vermilion border.

## QUESTIONS

1. **What is the most frequent malignant epithelial tumor of the lips?**
2. **Discuss the key principles of lower lip reconstruction.**
3. **How is the Camille Bernard flap designed and elevated?**
4. **The functional postoperative prognosis of lower lip reconstruction.**

## DISCUSSION

Lip cancer is the most common malignant lesion of the oral cavity, constituting 25% to 30% of all oral cavity cancer cases, and is the second most common malignancy of the head and neck overall (after cutaneous malignancy). Surgical therapy includes excision of the tumor with oncological margins of resection and reconstruction when it is necessary. Margins should be 0.5 cm for squamous cell carcinoma, as opposed to intraoral lesions, which require margins of 2 cm.^1^

There are a number of surgical options for reconstruction of the lower lip. A detailed analysis of the lip defect is mandatory before selecting the appropriate method of reconstruction.^2^ Lesions involving up to one-half of the lower lip can be excised and repaired primarily. Defects larger than one-half of the lip cannot be closed primarily without undue wound tension. Strategies for closure involve borrowing tissue either from the opposite lip or from the cheek using the Abbe, Karapandzic, or Estlander flap. Lesions involving from two-thirds to the entire lip are best reconstructed by using adjacent cheek tissue with the Camille Bernard flap. If the defect is larger than this, a free flap is used for reconstruction. It is important to adapt the reconstruction to the needs of the patient.^3^

The Camille Bernard flap is probably the most popular of all cheek advancement methods for subtotal full-thickness lower lip reconstruction. When possible during the excision of the lip lesion, a segment of labial mucosa is left attached along the labioalveolar groove centrally. This mucosa helps preserve the sulcus and allows a tension-free closure to the flap mucosa. Burow triangles are designed bilaterally, with each base equal in width to one-half of the lip defect. A line is first drawn horizontally and slightly superiorly from the oral commissure. Burow triangles are designed at their apexes at the nasolabial fold, their medial sides at the nasolabial fold, and their lateral sides connecting the apex to the horizontal line drawn from the commissure: The designed Burow triangles are then modified to a half-crescent configuration. The half-crescents of skin (Burow triangles) are then excised, preserving the underlying muscle. Facial musculature along the base of the triangle is incised as far laterally as necessary to suture the muscle in the midline. The mucosa is incised intraorally slightly superior to the incision through the muscle to preserve a cuff of mucosa to help reconstruct the vermilion border. Half-crescents of skin are also removed as needed from the lower portion of the chin at the chin-cheek junction. Intraoral mucosal advancement flaps are also created as diagramed to create a new lip. After closure, a portion of skin is excised and intraoral mucosa is advanced to create a new vermilion border.^4^

After surgery, patient satisfaction increased together with the improvement in lip competence, scar fading, and facial appearance. Critical to a functional lower lip reconstruction are sufficient oral access, preservation of sensation, oral competence, and muscle integrity.^5^ Using this technique, functional rehabilitation seems to be improved with muscle transposition. The distinct advantage of this operation is its ability to reconstruct nearly the entire lower lip in a single-stage procedure. An obvious disadvantage is reduction in the size of the orifice of the oral cavity and a “permanent smile” deformity of the lips, particularly in edentulous patients.^6^

Fortunately, lip cancer remains one of the most curable malignancies in the head and neck region. The 10-year survival rate can be as high as 98% and recurrence-free survival is more than 90%.^1^

## Figures and Tables

**Figure 1:**
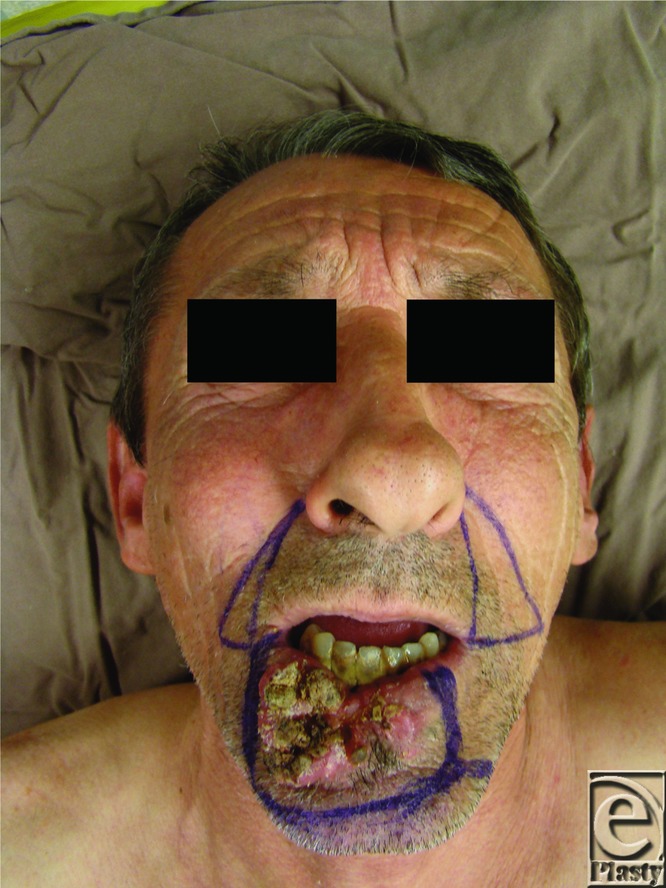
Preoperative aspect and the excisional pattern.

**Figure 2:**
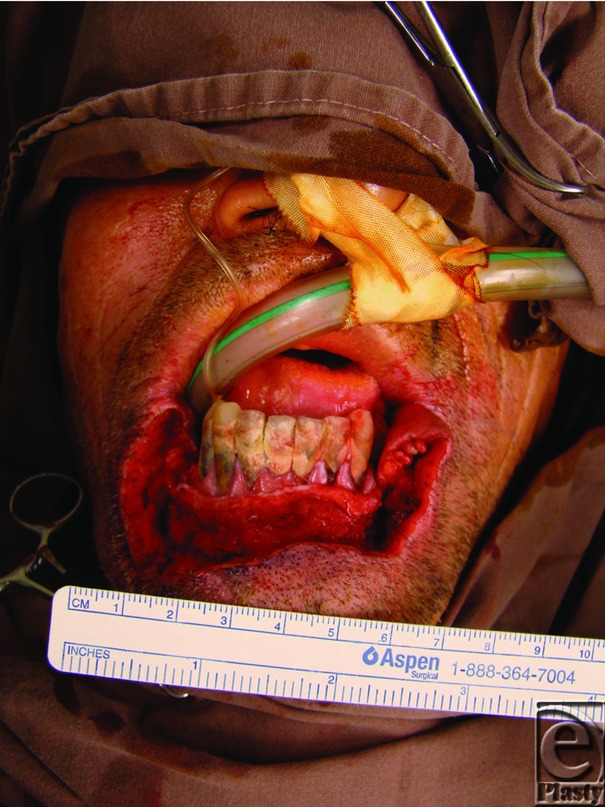
The defect result.

**Figure 3:**
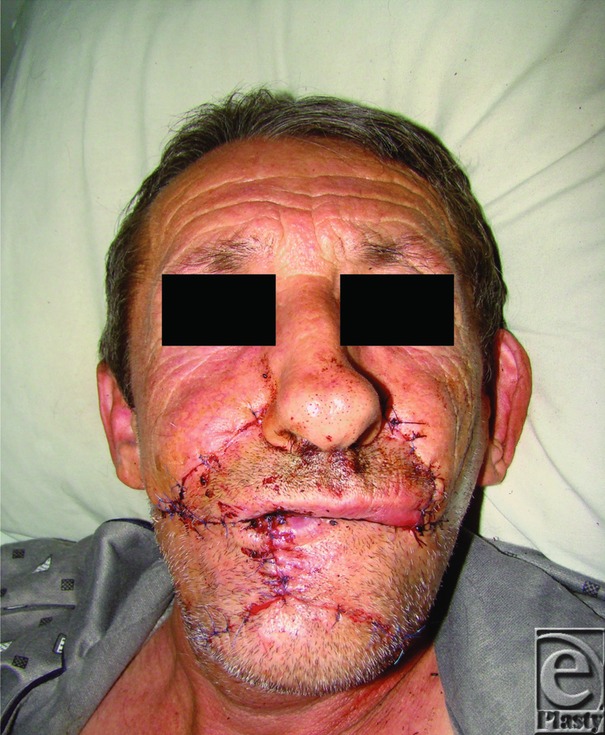
Immediate postoperative aspect.

**Figure 4:**
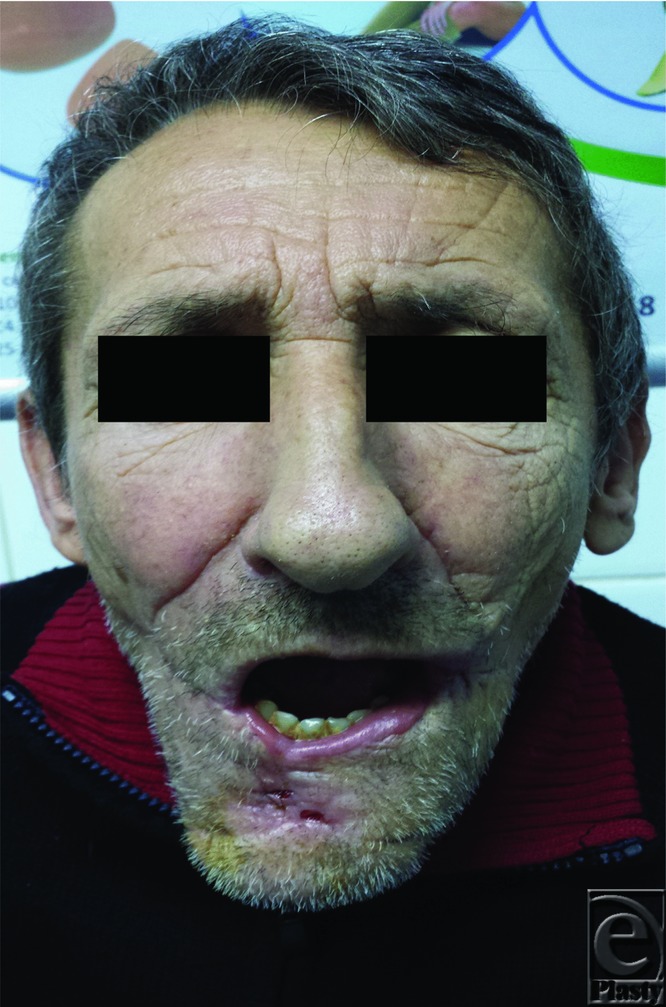
2-month postoperative aspect.
